# Supplementary material for: Association between oral microbiome and seven types of cancers in East Asian population: a two-sample Mendelian randomization analysis
Source: Front Mol Biosci. 2023 Nov 21;10:1327893. doi: 10.3389/fmolb.2023.1327893 (PMC10702768; doi:10.3389/fmolb.2023.1327893)
Supplement: Supplementary file 1 [file DataSheet1.ZIP › Supplementary Materials/MR plots of saliva/saliva═╝/Gastric cancer/pheno.1371_to_gastric cancer_forest.pdf]

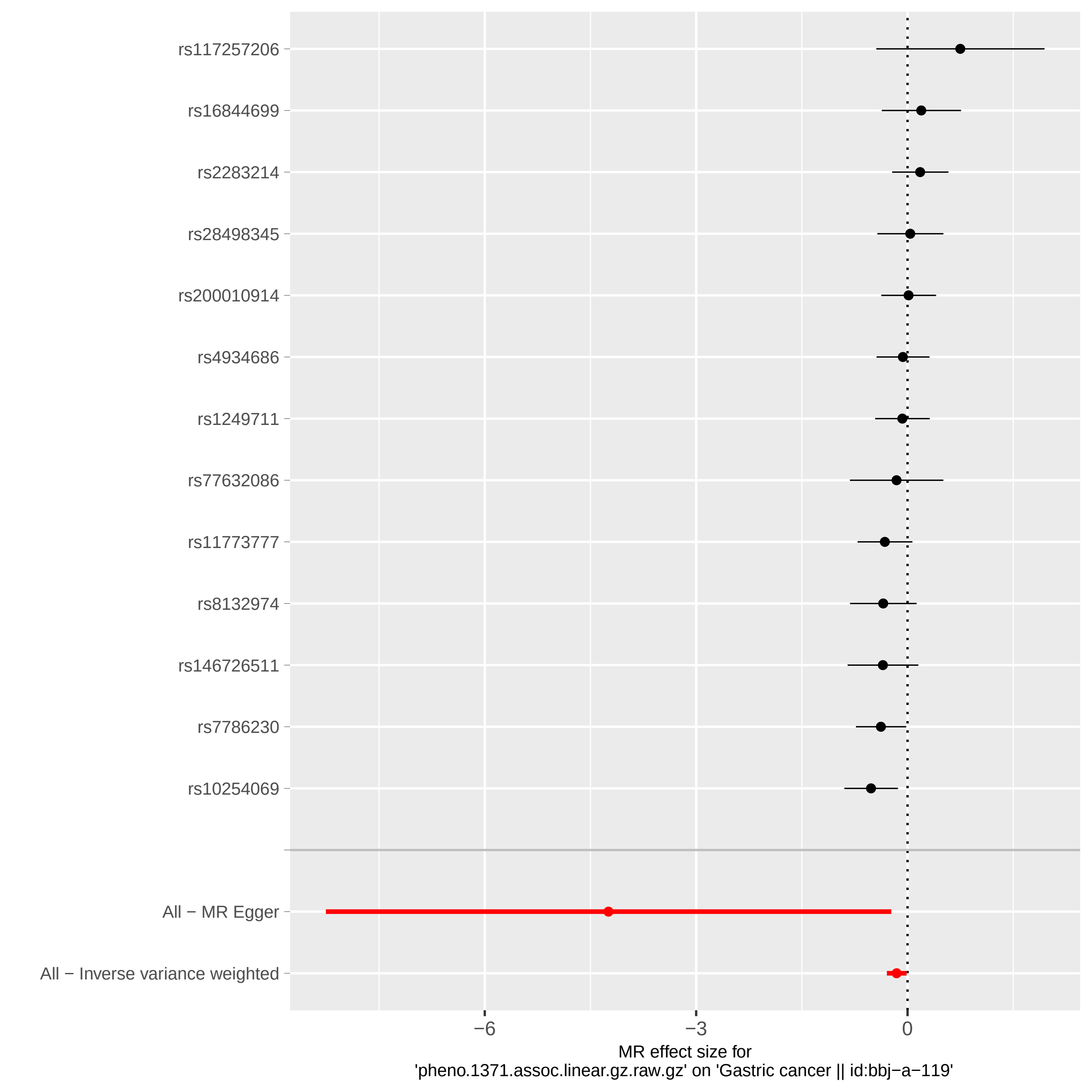

rs117257206
rs16844699
rs2283214
rs28498345
rs200010914
rs4934686
rs1249711
rs77632086
rs11773777
rs8132974
rs146726511
rs7786230
rs10254069
All − MR Egger
All − Inverse variance weighted
−6
−3
0
MR effect size for
'pheno.1371.assoc.linear.gz.raw.gz' on 'Gastric cancer || id:bbj−a−119'
